# Supplementary material for: Reduced ATP turnover during hibernation in relaxed skeletal muscle
Source: Nat Commun. 2025 Jan 2;16:80. doi: 10.1038/s41467-024-55565-4 (PMC11696273; doi:10.1038/s41467-024-55565-4)
Supplement: Supplementary file 1 — Supplementary Information [file 41467_2024_55565_MOESM1_ESM.pdf]

a

b

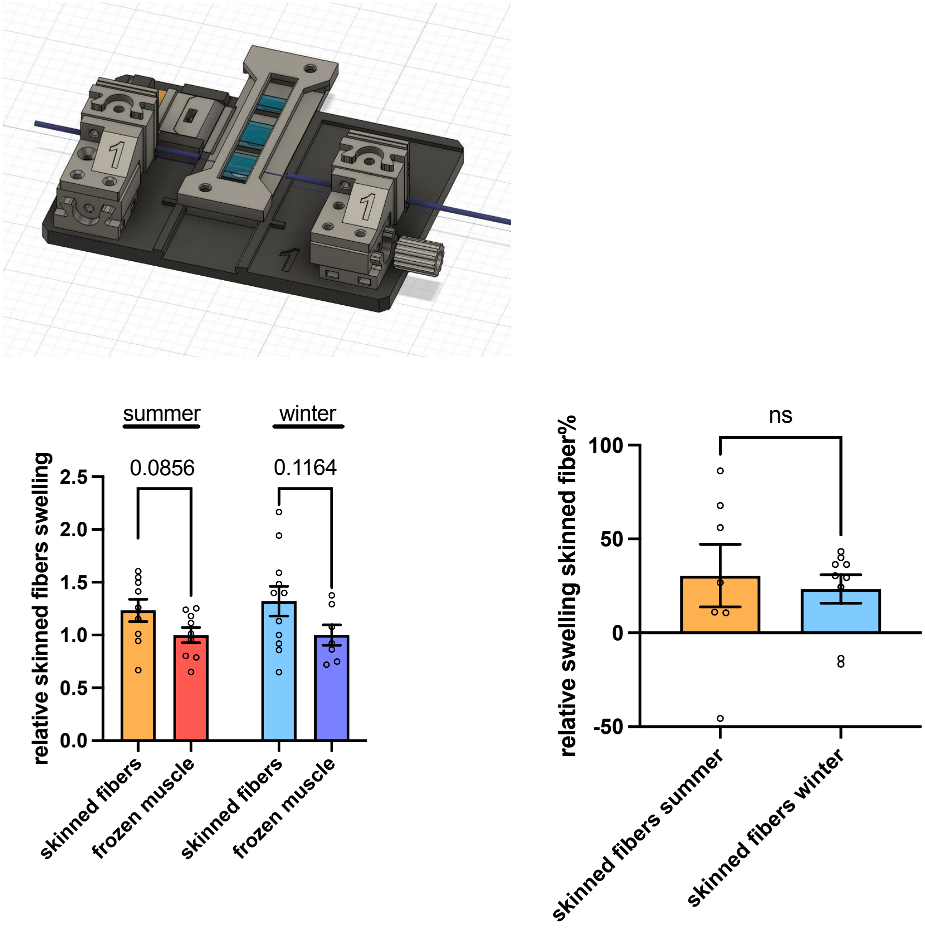

Supplementary figure 1. Schematic design of the 3d-printed setup used for mantATP chasing experiments and skinned fibers swelling evaluation. a Design of the 3D-printed incubation chamber used for the MantATP chasing experiments. **b** Swelling of skinned fibers is determined by comparing average cross-sectional area on frozen sections to the cross-sectional area determined for individual skinned fibers placed at optimal length. Swelling is clear for winter and summer fibers, without any difference between groups (n=7-11, Source data are provided as a Source Data file. mean±SEM; Unpaired t-test skinned fibers and frozen muscle. Mann-Whitney test skinned fibers summer and skinned fibers winter. P value= 0.8371). All data related to summer samples are reported in orange/red color, while those related to winter are in light blue/blue.

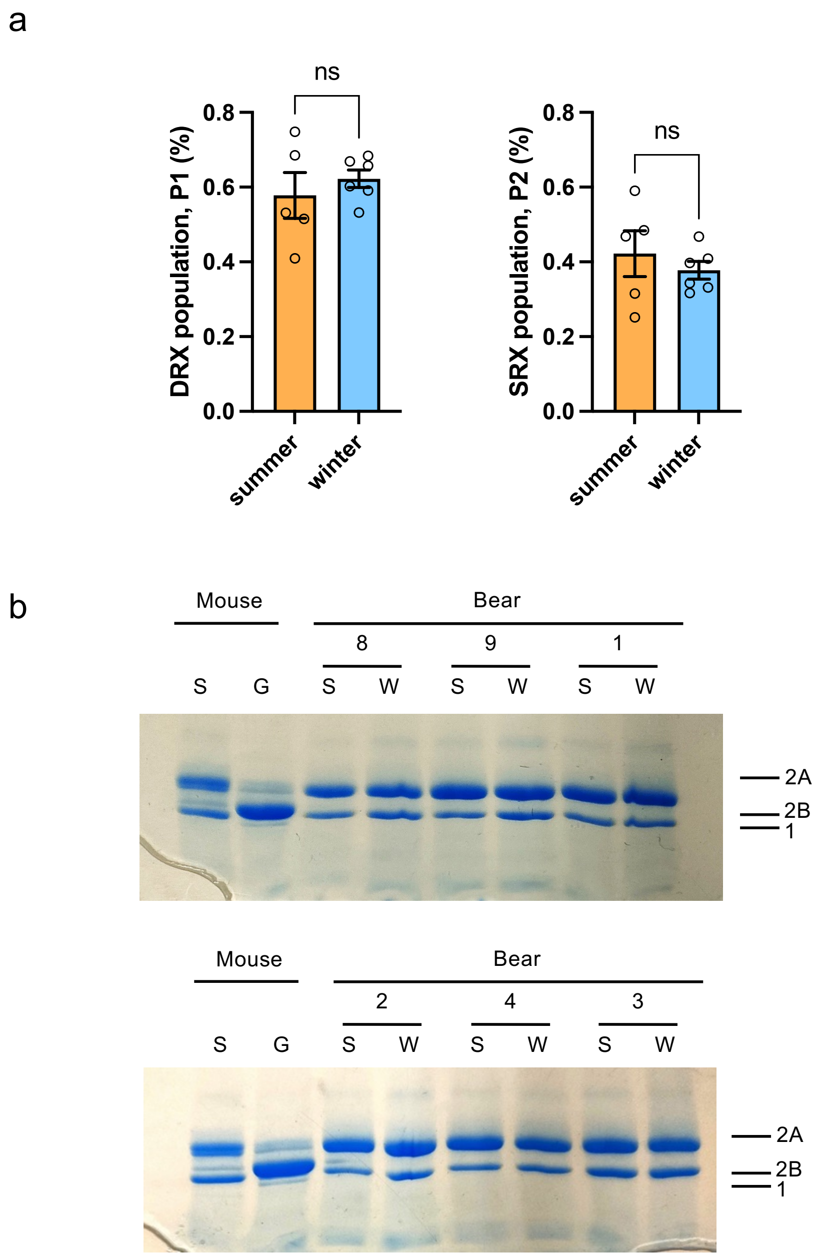

Supplementary figure 2. MantATP chasing population parameters and myosin isoform electrophoresis analysis of bear samples. **a** Relative population of myosin heads in DRX (P1. n=5-6; mean±SEM. Two-sided Unpaired t-test, P value=0.4834) or SRX (P2. n=5-6; mean±SEM. Two-sided unpaired t-test, P value=0.4834) in summer and winter biopsies show no differences. P1 and P2 are expressed as a percentage of P1 + P2, thus the total fitted populations, excluding the non-specific P0. All data related to summer samples are reported in orange, while those related to winter are in light blue. **b** Myosin heavy chain isoform composition in bear muscle analyzed on gel electrophoresis showed no major shift among summer and winter samples. On the left two mouse samples were loaded, with a slow (soleus, S) and fast (gastrocnemius, G) mouse muscle lysate to indicate the weight of the different isoforms (n=6 bears, n=6 summer and n=6 winter samples).

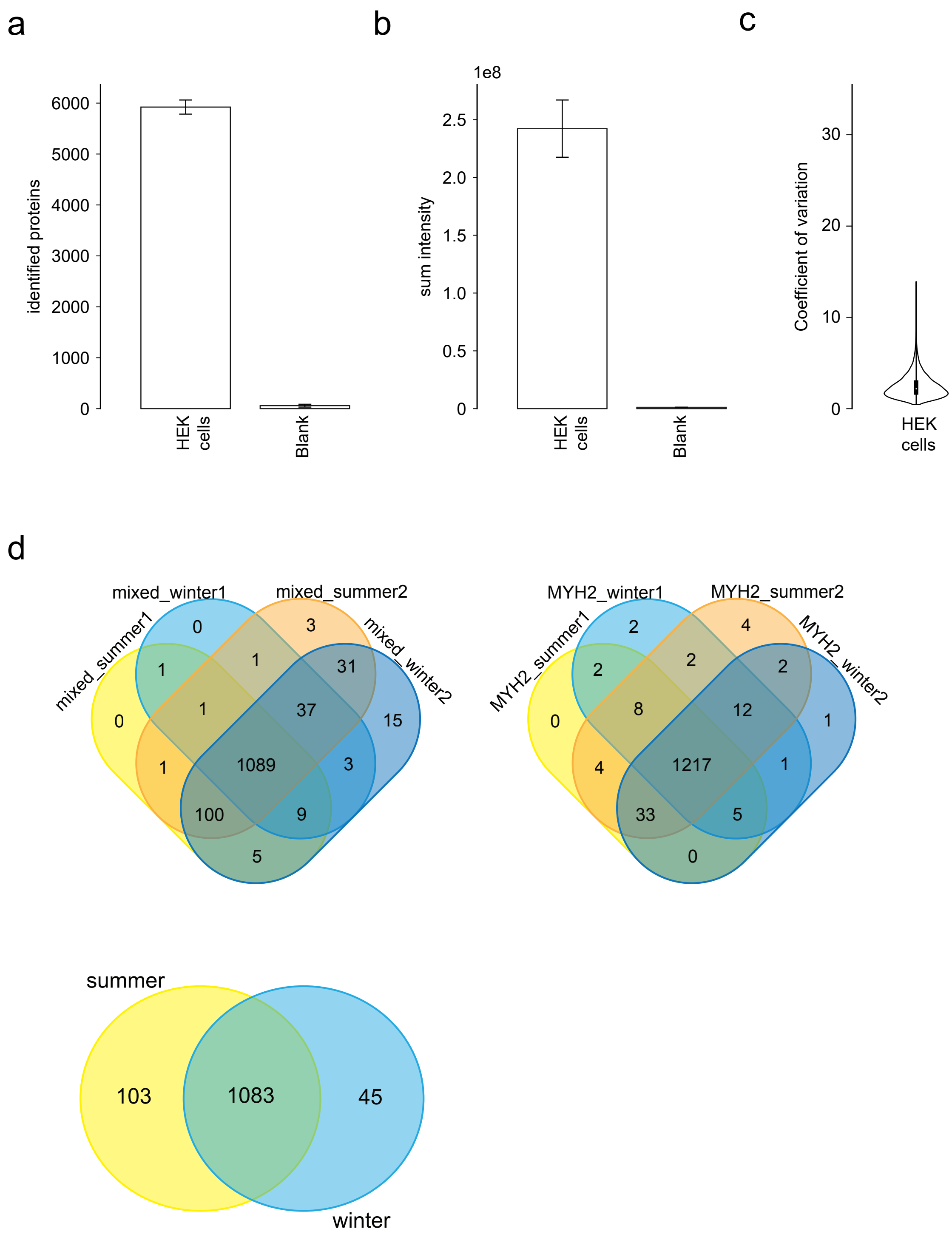

Supplementary figure 3. **Proteomic validation data. a,b and c** HEK cell lysates were analyzed as LC-MS performance control (CV of <5%) and blank controls were injected every 15 samples, identifying minimal protein carry-over per sample (<50). **d** Venn diagram showing the presence of specific proteins in the different groups. Most proteins are found in both winter and summer muscles.

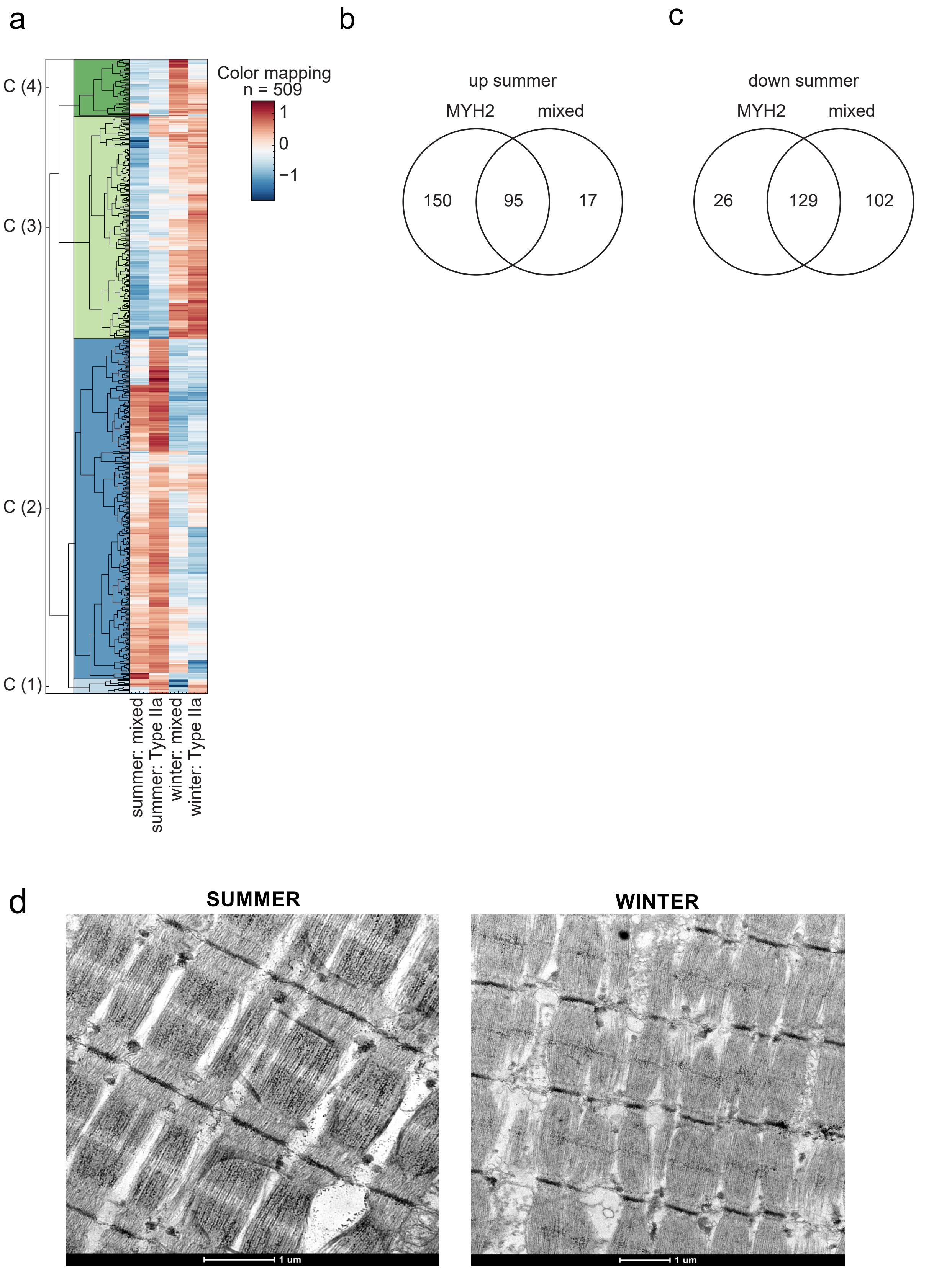

Supplementary figure 4. Clustering of proteomic data highlighting MYH2 and mixed fiber type protein content and electron microscopy picture of winter and summer biopsies. a Heat map showing hierarchical clustering of median normalized Z-scores for mixed fibers in both summers and winters. As can be seen, protein regulation is similar between the two different years. **b, c** Venn diagrams showing significantly up- and down-regulated proteins in fast MYH2 and mixed fibers. **d** Electron microscope images of skinned fibers from winter and summer fibers. Spaces are observed between sarcomeres in both cases, which are due to swelling.

Oxygen consumption: Resting summer = 0.276 ml g^{-1} h^{-1}
Hibernating winter = 0.069 ml g^{-1} h^{-1}
(Tøien et al. 2019)

Average bear body weight = 48.8 kg

1 ml oxygen = 0.005 kcal
(Scott 2005)

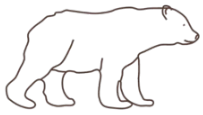

Daily energy expenditure:
Resting summer = 1940 kcal
Hibernating winter = 485 kcal

Resting skeletal muscle basal metabolism = 13 kcal/kg per day
(Elia et al. 1992)

Muscle mass (40% of BW) = 19.5 Kg

Muscle basal metabolism: Summer = 254 kcal
Winter = 64 kcal
(25% of summer calories)

ATPase activity: Summer = 0.052 ATP*s^{-1}
Winter = 0.032 ATP*s^{-1}

1 ATP = 7.3 kcal/mol

Skeletal muscle efficiency 0.5
(Zhen-He He et al. 2000)

6.06E-24 kcal/ ATP molecule

Summer
2.7E-20 kcal/day

Winter
1.68E-20 kcal/day

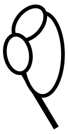

per single myosin

Average bear body weight = 48.8 kg

Muscle mass (40% of BW) = 19.5 Kg

Myosin concentration in vivo = 150 μM
(Ferenczi et al. 1984)

Total myosin molecules = 1.7E21

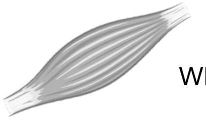

Whole muscle energy consumption due to resting myosin: Summer = 48 kcal
Winter = 29.5 kcal

	Daily energy expenditure (kcal)	Skeletal muscle basal metabolism (kcal)	Resting myosin energy consumption (kcal)	Resting myosin contribution
Summer	1940	254	48	18.9% of muscle tissue, 2.5% of whole body energy expenditure
Winter	485	64	29.5	46.6% of muscle tissue, 6% of whole body energy expenditure

Supplementary figure 5. Estimation of the energetic contribution of relaxed myosin ATP consumption on the tissue basal metabolism and whole animal energy expenditure.

If we multiply the rates obtained by our resting ATPase activity (0.052 ATP/s^{-1} during active season and 0.032 ATP/s^{-1} during hibernation) for a whole day we obtain the consumption of 4493 ATP during active season and 2765 ATP during hibernation, values normalized for a single myosin head. The caloric contribution of 1 mole of ATP is estimated to be 7.3 kcal/mol. An efficiency of 0.4 has been reported for skeletal muscle during contraction [1], in these calculations an efficiency of 0.5 has been assumed, since no mechanical force is exerted. It is possible now convert the consumption of ATP in energetic contribution of a single myosin head, being that 2.7E-20 kcal for summer biopsies and 1.68E-20 kcal for hibernating muscle. To estimate the number of myosin head we can apply the in vivo myosin concentration of 150µM[2] to the muscle mass estimated for an average bear in our study which is 19.5Kg (40% of the average body weight during active period which states at 48.8 Kg). The total number of myosin heads is estimated to be 1.7E21. Now it is possible to multiply the energetic contribution of a single myosin head for the number of heads, obtaining the overall energy contribution of 48 kcal/day for active season and 29.6 kcal/day during hibernation. For a bear of average weight of those reported in this work (48.8 Kg during active period), the estimated DEE is about 1940 kcal/day in the active season, and it goes down to 485 kcal/day during hibernation. These values were obtained by considering the active oxygen consumption of 0.276 ml g^{-1} h^{-1} and an oxygen consumption during hibernation of 0.069 ml g^{-1} h^{-1} [3] The conversion was calculated considering the use of 1L of oxygen to obtain 5 kcal[4]. From measurements on human tissues, resting skeletal muscle energy expenditure is estimated to be 13 kcal/kg*day [5] and in our case is applied to a bear muscle mass of about 19.5Kg as stated earlier. Thus, resting muscle energy expenditure contributes to about 254 kcal/day in active season, and 63.5 kcal/day during hibernation (assuming a decrease to 25%[3]). Combining the two results we obtain a contribution of myosin resting state which accounts for 18% of the tissue DEE (48kcal over 254kcal) during summer, and 47% of tissue DEE during winter (30kcal over 63.5kcal). So, based on ATPase results, the energy sparing of hibernating muscle compared to active is estimated to be 38%.

This rough esteem could not consider the possible interaction of shivering events and active muscle movements which could potentially induce a temporary shift of myosin heads towards a more disordered state, populating DRX in vivo. Moreover, our analysis was performed at a temperature that is below the physiological, and these calculations were done without accounting for differences in body temperature during activity and hibernation. Nevertheless, we found a convergent estimation on the energy saving extent applying both mantATP chasing and combined ATPase rates, stating respectively 28% and 38%. Despite having profound limitations, this estimation leads to the idea that an energy sparing contribution of myosin SRX exists, and it could be in the range of 30%.

Most of the literature regarding metabolism and energy utilization of skeletal muscle cells is focused on the balance and rearrangements during exercise[6] to supply energy to excitation-contraction coupling and contracting myofilaments. The available resources covering energetic management in resting muscle tissue are very limited, thus, our estimation needs to fill up the blanks with reasonable guesses. According to our chasing results, the difference in time constant between myosin DRX and SRX leads to a theoretical decrease in ATP consumption which is of about 25% in disordered myosin heads population and 39% due to super relaxed myosin comparing summer and winter samples. Assuming no significant difference in populations entity, the energy saving of the two populations combined can be estimated as a 28% reduction in biopsies of hibernating bears compared to active ones. Figure has been built using Microsoft Powerpoint, bear figure in top right corner has been kindly provided by L. Cussonneau, single myosin head schematic was drawn using Powerpoint, muscle figure has been obtained from Biorender (licensed to LN, Department of Biomedical Science, University of Padova).

References

1. He, Z. H., Bottinelli, R., Pellegrino, M. A., Ferenczi, M. A. & Reggiani, C. ATP consumption and efficiency of human single muscle fibers with different myosin isoform composition. *Biophys J* 79, 945–961 (2000).
2. Ferenczi, M. A., Homsher, E., Simmons, R. M. & Trentham, D. R. Reaction mechanism of the magnesium ion-dependent adenosine triphosphatase of frog muscle myosin and subfragment 1. *Biochemical Journal* 171, 165–175 (1978).
3. Tøien, Ø. *et al.* Hibernation in Black Bears: Independence of Metabolic Suppression from Body Temperature. *Science* 331, 906–909 (2011).
4. Scott, C. Misconceptions about Aerobic and Anaerobic Energy Expenditure. *J Int Soc Sports Nutr* 2, 32–37 (2005).
5. Elia, M. Organ and tissue contribution to metabolic rate. *Energy Metabolism, Tissue Determinants and Cellular Corollaries* 61–80 (1992).
6. Frøbert, A. M. *et al.* Differential Changes in Circulating Steroid Hormones in Hibernating Brown Bears: Preliminary Conclusions and Caveats. *Physiol Biochem Zool* 95, 365–378 (2022).
